# Effect of gonadotropin-releasing hormone agonist monotherapy and combination therapy with growth hormone on final adult height in girls with central precocious puberty

**DOI:** 10.1038/s41598-023-28602-3

**Published:** 2023-01-23

**Authors:** Ah Young Cho, Young Suk Shim, Hae Sang Lee, Jin Soon Hwang

**Affiliations:** Department of Pediatrics, Ajou University School of Medicine, Ajou University Hospital, San 5, Wonchon-dong, Yeongtong-gu, Suwon, 443-721 Korea

**Keywords:** Endocrinology, Medical research

## Abstract

This study aimed to compare clinical parameters, including final adult height (FAH), in girls with central precocious puberty treated with gonadotropin-releasing hormone agonists (GnRHa) with and without growth hormone (GH). This retrospective study reviewed data of 210 girls with precocious puberty who had reached FAH in a long-term trial of GnRHa treatment. The subjects were divided into the GnRHa treatment group (n = 188), and the combined GnRHa + GH treatment group (n = 22). Chronological age, bone age, height, height standard deviation score, predicted adult height (PAH), FAH, Tanner stage, and hormone levels were assessed during the treatment period. At the start of treatment, PAH was 156.35 ± 6.34 cm in the GnRHa monotherapy group and 150.41 ± 5.32 cm in the GnRHa + GH group (*P* < 0.001). At the end of treatment, PAH was 166.25 ± 5.26 cm in the GnRHa group and 164.07 ± 4.99 cm in the combined GnRHa + GH treatment group, which had increased compared to the start of treatment. The FAH in the GnRHa group and GnRHa + GH combination group were 161.07 ± 4.78 cm and 159.63 ± 3.8 6 cm, respectively, without significant difference. In addition, the height gain (FAH–PAH) was significantly higher in the GnRHa + GH group than the GnRHa group (9.22 ± 6.03 cm vs. 4.72 ± 5.01 cm, *P* < 0.001). In girls with central precocious puberty, the height gain in the FAH compared to PAH at the start of treatment was significantly higher with the GnRHa + GH combination treatment.

## Introduction

Central precocious puberty (CPP) refers to conditions in which secondary sexual characteristics develop before the age of 8 years in girls and before the age of 9 years in boys due to activation of the hypothalamic-pituitary–gonadal axis^1,2^. Early puberty accelerates growth and promotes bone maturation, resulting in early fusion that causes a decrease in final adult height (FAH)^3–5^. Gonadotropin-releasing hormone agonists (GnRHa) including leuprolide acetate and triptorelin have been used as a standard treatment for CPP for decades to suppress the secretion of sex hormones, inhibit rapid bone maturation, and extend the growth period, thereby improving FAH^5–8^. However, several reports suggest that GnRHa treatment can reduce the growth rate below the age-appropriate normal range, and that this phenomenon may be associated with a decrease in the biological activity of insulin-like growth factor-1 (IGF-1) levels^9–11^. Therefore, in recent clinical trials, if the predicted adult height (PAH) is small or the height velocity is decreased among the children who have been treated with GnRHa, growth hormone (GH) is added to GnRHa to compensate for the decreased IGF-1.

In this study, we aimed to analyze the effect on FAH and the change in PAH in girls diagnosed with CPP by classifying them into two groups; those that received GnRHa monotherapy and those that received combined GnRHa + GH treatment.

## Materials and methods

### Study design and patients

This was a retrospective study of 210 girls diagnosed with and treated for CPP between January 2006 and April 2021 at the Department of Pediatrics of Ajou University Hospital, whose FAH could be assessed in a long- term trial of GnRHa treatment. CPP affects girls five to ten times more frequently than it does boys^12^. Therefore, only girls were enrolled in this study. The subjects were divided into the GnRHa monotreatment group (n = 188), and the combined GnRHa + GH treatment group (n = 22). GH treatment was recommended when the PAH at the start of therapy was less than -2 standard deviation score (SDS) than the target height (TH) or the growth rate was less than 4 cm/year.

In the GnRHa monotreatment group, the average duration of GnRHa treatment was 3.21 ± 0.73 years. In addition, the GnRHa + GH treatment group’s mean GnRHa treatment duration was 3.00 ± 0.58 years. GH treatment was added after an average of 6.9 months after starting GnRHa treatment. The mean duration of total GH treatment was 2.99 ± 1.25 years and that of the combined treatment was 2.04 ± 0.90 years. Out of 22 patients with GnRHa + GH treatment, GH treatment was stopped in 13 patients upon completion of GnRHa treatment, and GH treatment was continued in 9 patients even after discontinuation of GnRHa treatment.

Chronological age (CA), bone age (BA), BA SDS, BA–CA, height, height SDS, weight, weight SDS, body mass index (BMI), BMI SDS, PAH, PAH SDS, luteinizing hormone (LH) level, and follicle-stimulating hormone (FSH) level were measured at three time points: the start of GnRHa treatment, after 1 year of treatment, and at the end of treatment.

The diagnostic criteria for idiopathic CPP are (1) breast enlargement before 8 years of age, (2) LH levels (cutoff: ≥ 5 IU/L) in response to the GnRH stimulation test, and (3) bone age advance greater than 1 year above chronological age^13^. We excluded girls with brain tumors or ovarian or adrenal lesions. In addition, thyroxine and thyroid stimulating hormone (TSH) levels were measured in all patients to exclude hyperthyroidism.

Leuprolide acetate was administered every 28 days at a dose of 3.75 mg in girls weighing more than 30 kg, 2.5 mg in girls weighing between 20 and 30 kg, and 1.87 mg in girls weighing less than 20 kg.

The initial dose of GH treatment in all patients was 0.6 IU/kg/wk. The GH dose was divided and administered subcutaneously 6 times a week.

The present study was approved by the Institutional Review Board of the Ajou University Hospital (AJIRB-MED-MDB-19-538) and was exempted from informed consent requirements owing to its retrospective design; all measurements were performed as part of routine practice. The study adhered to the tenets of the Declaration of Helsinki.

### Measurements

BA was measured using the by taking Greulich–Pyle method simple radiographs of the left hand and wrist^14^. TH was defined as the midparental height, which was calculated by subtracting 6.5 cm to the average parental height. The Bayley–Pinneau (BP) advanced table was used to measure PAH^15^. The FAH was defined as the height measured when BA reached 15 years or growth rate was < 1 cm/year. The standard growth chart for children and adolescents published by the Korean Pediatrics Association in 2017 was used^16^. Serum LH and FSH levels were measured by IRMA (BioSource, Nivelles, Belgium). The detection limits for the LH and FSH assays were 0.1 IU/L and 0.2 IU/L, the intra-assay coefficients of variation (CV) were 1.4–3.9% and 1.1–2.0%, and the interassay CVs were 3.4–8.0% and 2.4–4.4%, respectively.

### Statistical analysis

All statistical analysis were performed using SPSS version 24 (SPSS Inc. Chicago, USA) and the results were expressed as the mean value ± standard deviation. An independent t-test was performed to evaluate the significance of the difference in auxological and biochemical factors between the GnRHa group (n = 188) and the GnRHa + GH group (n = 22). A comparative analysis between the two groups was conducted before treatment with GnRHa, after 1 year of treatment with GnRHa, at the end of treatment with GnRHa, and when final adult height was reached. For factors that can affect the height gain (FAH − initial PAH), simple linear regression and multiple linear regression with stepwise variable selection were used.

## Results

### Auxological and clinical characteristics of girls with idiopathic CPP according to type of treatment at the start of treatment

Clinical factors according to the type of treatment in a total of 210 girls are shown in Table 1.

**Table 1:** Auxological and clinical characteristics of the patients by type of treatment.

Variable	GnRHa (n = 188)	GnRH + GH (n = 22)	*P*-value
CA (year)	8.20 ± 0.62	8.34 ± 0.44	0.298
BA (year)	10.30 ± 0.77	10.51 ± 0.61	0.198
BA (SDS)	3.59 ± 1.12	4.20 ± 1.21	0.020
BA–CA	2.10 ± 0.72	2.18 ± 0.73	0.634
Tanner stage (breast)			0.550
II	130 (69.1%)	16 (72.7%)	
III	31 (58.5%)	88 (65.7%)	
IV	7 (2.1%)	0	
Tanner stage (pubic hair)			0.552
I	184 (97.9%)	22 (100.00%)	
II	4(2.1%)		
Height	132.24 ± 5.17	128.52 ± 3.84	< 0.001
Height SDS	1.10 ± 0.79	0.32 ± 0.73	< 0.001
Weight (Kg)	30.94 ± 5.34	28.55 ± 4.00	0.043
Weight SDS	0.65 ± 3.25	0.37 ± 0.73	0.693
BMI (kg/m^2^)	17.64 ± 2.50	17.25 ± 1.73	0.479
BMI SDS	− 0.28 ± 10.63	0.29 ± 0.81	0.479
PAH (cm)	156.35 ± 6.34	150.41 ± 5.32	< 0.001
PAH SDS	− 0.92 ± 1.31	− 2.17 ± 1.17	< 0.001
TH (cm)	159.01 ± 3.69	158.93 ± 4.01	0.923
Basal LH (mIU/mL)	1.07 ± 0.86	1.15 ± 1.41	0.787
Basal FSH (mIU/mL)	2.50 ± 1.74	2.60 ± 1.29	0.796
Peak LH (mIU/mL)	11.89 ± 11.14	11.78 ± 9.57	0.964
Peak FSH (mIU/mL)	12.96 ± 4.39	13.46 ± 4.21	0.615
Duration of GnRHa treatment(year)	3.21 ± 0.73	3.00 ± 0.58	0.184
Time of menarche after GnRHa treatment (year)	1.34 ± 0.51	1.17 ± 0.53	0.145
Menarche age(year)	12.74 ± 0.60	12.50 ± 0.60	0.085
Duration of GH treatment after GnRHa treatment (months)		6.59 ± 7.77	
Duration of total GH treatment (years)		2.99 ± 1.25	

*GnRHa* Gonadotropin-releasing hormone agonist, *GH* Growth hormone, *CA* Chronological age, *BA* Bone age, *SDS* Standard deviation score, *BMI* Body mass index, *PAH* Predicted adult height-calculated with advanced table of baley–Pinneau(BP)method, *TH* Target height, *LH* Luthenizing hormone, *FSH* Follicular stimulating hormone.

In the GnRHa group, the CA and BA at the start of treatment were 8.20 ± 0.62 years and 10.30 ± 0.77 years, respectively. In the GnRH + GH group, the CA and BA at the start of treatment were 8.34 ± 0.44 years and 10.51 ± 0.61 years, respectively. There was no significant difference in BA and CA in both groups, but BA SDS was significantly higher in the GnRHa + GH group than in the GnRHa group (4.20 ± 1.21 vs. 3.59 ± 1.21, P < 0.05). The height and height SDS at start of treatment was significantly higher in the monotherapy group (132.24 ± 5.17 cm vs. 128.52 ± 3.84 cm, 1.10 ± 0.79 vs. 0.32 ± 0.79, respectively, *P* < 0.001).

**Figure 1:**
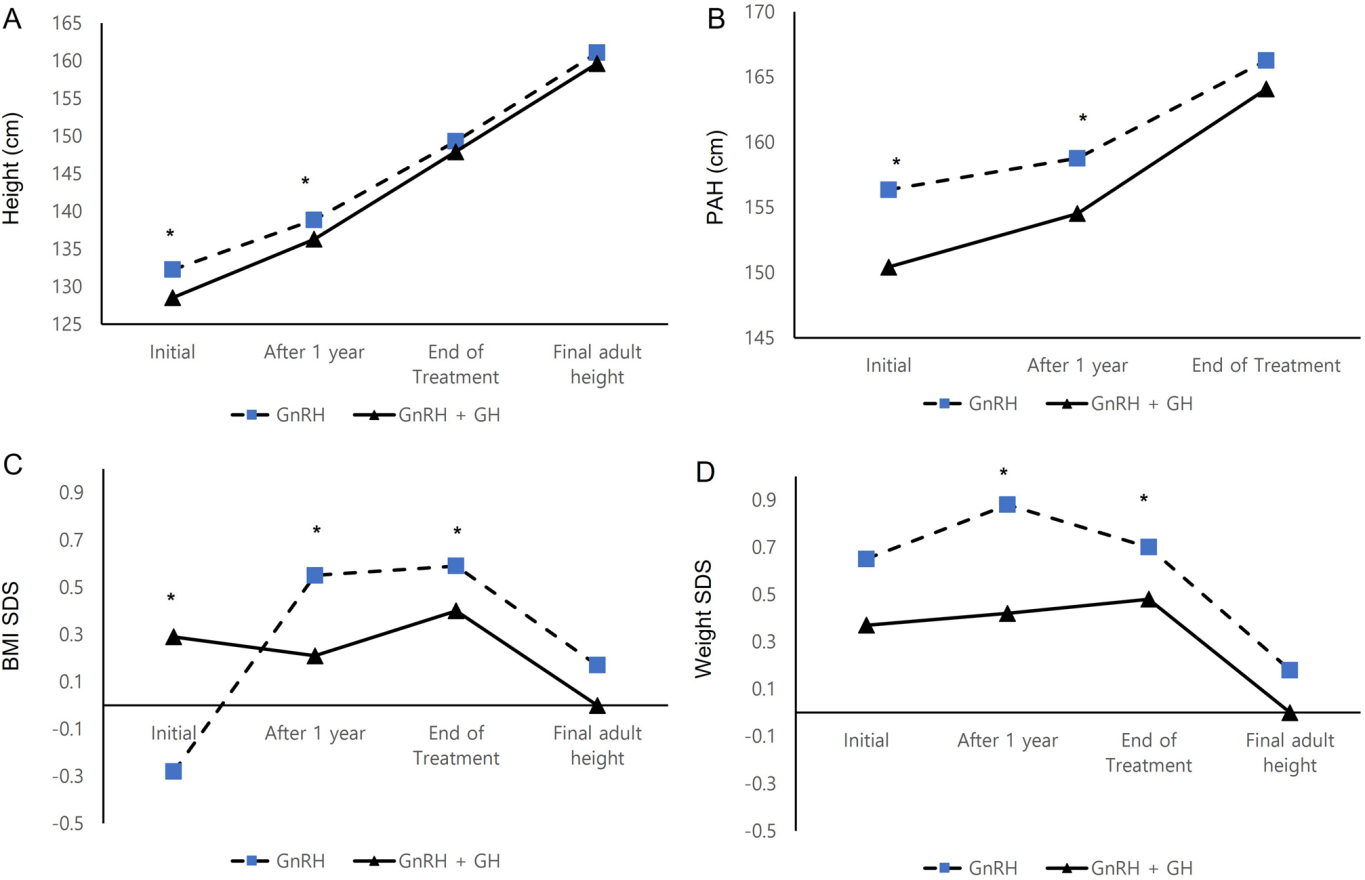
Comparison of height, PAH, BMI SDS and Weight SDS during treatment periods. *PAH* Predicted adult height, *BMI* Body mass index, *SDS* Standard deviation score. *P < 0.05 between GnRHa group and the GnRHa plus GH group.

At start of treatment, the PAH was 156.35 ± 6.34 cm in the monotherapy group and 150.41 ± 5.32 cm in the combination treatment group (Fig. 1); PAH was significantly lower in the combination group (*P* < 0.001). The TH was 158.93 ± 4.01 cm in the GnRHa + GH group, which was smaller than in the GnRHa group (159.0.1 ± 3.69 cm), but the was not significant. No patients with combined GH treatment had significant side effects including abnormal glucose metabolism, tumor development, or thyroid hormone abnormalities.

### Changes in auxological parameters after GnRHa treatment and GnRHa + GH treatment

We compared the results of auxological data between two groups during the treatment period (Table 2). In both groups, it was confirmed that PAH and PAH SDS increased after 1 year of treatment and at the end of treatment compared to at the start of treatment, indicating that growth potential was restored. After 1 year of treatment, the PAH and PAH SDS were significantly higher in GnRHa monotherapy group than the GnRH + GH combination group (158.76 ± 5.29 cm vs. 154.52 ± 4.73 cm, − 0.42 ± 1.06 vs. − 1.29 ± 0.99, respectively *P* < 0.001). At the end of treatment, the PAH and PAH SDS were 166.25 ± 5.26 cm and 1.01 ± 1.00 in the monotherapy group and 164.07 ± 4.99 cm and 0.63 ± 0.95 in the combination group, respectively, with no significant difference (Fig. 1). This indicates a large progression in PAH in the combination treatment group and improvement of height potential in the GnRHa + GH treatment group.

**Table 2:** Changes in auxological parameter after GnRHa treatment or GnRHa + GH combination treatment.

Variable	GnRHa (n = 188)	GnRHa + GH (n = 22)	*P*-value
1 year after treatment			
Height(cm)	138.84 ± 5.00	136.29 ± 4.27	0.023
Height SDS	1.06 ± 0.72	0.60 ± 0.83	0.006
PAH (cm)	158.76 ± 5.29	154.52 ± 4.73	< 0.001
PAH SDS	−0.42 ± 1.06	−1.29 ± 0.99	< 0.001
At end of treatment			
Height (cm)	149.28 ± 5.02	147.89 ± 4.83	0.346
Height SDS	0.50 ± 0.77	0.37 ± 0.73	0.278
PAH (cm)	166.25 ± 5.26	164.07 ± 4.99	0.067
PAH SDS	1.04 ± 1.00	0.63 ± 0.95	0.068
At final adult height			
FAH (cm)	161.07 ± 4.78	159.63 ± 3.86	0.174
Height SDS	−0.09 ± 0.94	−0.23 ± 0.77	0.530

*GnRHa* Gonadotropin-releasing hormone agonist, *GH* Growth hormone, *CA* Chronological age, *BA* Bone age, *SDS* Standard deviation score, *BMI* Body mass index, *PAH* Predicted adult height-calculated with advanced table of baley–Pinneau (BP) method, *FAH* Final adult height.

### Height outcome in CPP with GnRHa treatment or GnRHa + GH treatment

The height gain (FAH–initial PAH) in the GnRHa + GH group was 9.22 ± 6.03 cm, which was significantly higher than in the GnRHa group (4.72 ± 5.01 cm, *P* < 0.001) (Table 3). In addition, in the treatment period, ΔPAH was significant higher in the GnRHa + GH group than in the GnRHa group (13.66 ± 6.39 cm vs. 9.90 ± 5.86 cm, *P* = 0.005). The total growth from the end of GnRHa therapy to FAH in GnRHa and GnRH + GH group was 11.79 ± 5.86 cm and 11.74 ± 2.70 cm, respectively, but there was no significant difference.

**Table 3:** Height outcome in subjects with GnRHa treatment or GnRHa + GH treatment.

Variable	GnRHa (n = 188)	GnRH + GH (n = 22)	*P*-value
During treatment			
Δ PAH*	9.90 ± 5.86	13.66 ± 6.39	0.005
Growth velocity (cm/year)	5.29 ± 5.86	6.40 ± 0.85	< 0.001
From end of GnRHa to final adult height			
Total growth (cm)	11.79 ± 5.86	11.74 ± 2.70	0.937
Final adult height			
Height gain (FAH- initial PAH) cm	4.72 ± 5.01	9.22 ± 6.03	< 0.001
Genetic height gain (FAH-TH) cm	2.04 ± 4.75	0.69 ± 3.53	0.199

*GnRHa* Gonadotropin-releasing hormone agonist, *GH* Growth hormone, *PAH* predicted adult height-calculated with advanced table of baley–Pinneau(BP)method, *FAH* Final adult height, *TH* Target height.

*Δ PAH: the difference between PAH at the end of treatment and initial PAH.

### Clinical factors influencing height gain (FAH − initial PAH)

We performed a simple linear regression analysis on the factors that influence the height gain (FAH − initial PAH) in subjects receiving GnRHa + GH treatment (Table 4). At the start of treatment, BA, BA SDS, PAH, and PAH SDS were correlated with height gain (β = 0.693, 0.600, − 0.775, − 0.769, respectively, all *P* < 0.05). After 1 year of treatment, BA, BA − CA, LH, and FSH had significant correlations with height gain (β  = 0.632, 0.484, − 0.533, − 0.485, respectively, all *P* < 0.05). At the end of treatment, there was no significant variable affecting the height gain. Furthermore, the duration and dose of GH treatment had no significant correlation with height gain. In the multiple linear regression analysis using stepwise variable selection based on factors that were significantly correlated with the height gain, the PAH and BA at start of treatment were found to be significantly correlated with FAH (Table 5).

**Table 4:** Factors influencing height gain in GnRHa + GH treated girls at GnRHa treatment.

	At GnRHa treatment		At 1 year after treatment		At end of treatment	
	Coefficient (*β*)	*Ρ*	Coefficient (*β*)	*Ρ*	Coefficient (*β*)	*Ρ*
TH (cm)	0.361	0.099				
CA (year)	− 0.128	0.571	− 0.125	0.581	0.264	0.235
BA (year)	0.693	< 0.001	0.632	0.002	0.155	0.539
BA–CA	0.600	< 0.001	0.484	0.023	0.141	0.576
Height	− 0.121	0.590	0.006	0.979	0.386	0.076
Height SDS	− 0.181	0.858	0.061	0.786	0.197	0.381
Weight	0.060	0.792	0.112	0.619	0.407	0.060
Weight SDS	0.121	0.592	0.157	0.485	0.304	0.169
BMI (kg/m^2^)	0.154	0.495	0.152	0.501	0.315	0.153
BMI SDS	0.168	0.456	0.172	0.445	0.274	0.216
PAH (cm)	− 0.775	< 0.001	− 0.295	0.183	0.325	0.140
PAH SDS	− 0.769	< 0.001	− 0.302	0.172	0.333	0.130
LH (mIU/mL)	− 0.375	0.085	− 0.533	0.023	− 0.029	0.911
FSH (mIU/mL)	− 0.128	0.571	− 0.485	0.041	− 0.397	0.114
E_2_ (pg/mL)	0.216	0.373	0.123	0.627	0.277	0.299
Dose of GH					− 0.338	0.124
Duration of GH treatment					0.371	0.090

*GnRHa* Gonadotropin-releasing hormone agonist, *GH* Growth hormone, *TH* Target height, *CA* Chronological age, *BA* Bone age, *SDS* Standard deviation score, *BMI* body Mass index, *PAH* Predicted adult height-calculated with advanced table of baley–Pinneau (BP) method; luthenizing hormone, *FSH* Follicular stimulating hormone; E_2_, estrogen.

**Table 5:** Multiple regression analysis of factors influencing height gain (FAH–initial PAH) in treated girls. (n = 22, R^2^ = 0.764).

Variable	*B*	SE	*P*
PAH at start of treatment(cm)	− 0.593	0.153	0.001
BA at start of treatment	0.394	1.344	0.019

Stepwise regression linear regression of the following independent variable: BA, BA–CA, PAH, PAH SDS, 1 yr BA, 1 yr BA-CA ,1 yr LH, 1 yr FSH.

## Discussion

In this study, the height gain, which is the difference between FAH and PAH at the start of treatment, was significantly higher in the GnRHa + GH group compared to the GnRHa group in girls with CPP.

GnRHa is a standard treatment for CPP in both boys and girls. It is known to suppress the secretion of sex hormones to slow puberty and suppress rapid bone fusion to achieve FAH within the TH range^17,18^. However, several studies have reported that GnRHa can reduce the growth velocity below the normal range suitable for age^7,19,20^. Studies on changes in the GH-IGF-1 axis during GnRHa treatment have been conducted; although no consensus has been reached, some studies have reported that a decrease in the biologically active IGF-1 level contributes to the subnormal growth velocity^8,10,21,22^. Therefore, in the current clinical trial, if the PAH was very short or the growth rate showed a noticeable reduction during GnRHa treatment, GH combination treatment was used to improve FAH.

In a previous study that analyzed factors affecting the subnormal growth velocity during GnRHa treatment in 50 girls with idiopathic CPP^23^, the average age when subnormal growth velocity appeared was 9.9 years. In addition, the third year of GnRHa treatment carried the highest risk of subnormal growth velocity. There was a significant negative correlation with growth velocity SDS in the third and fourth years of treatment with BA at diagnosis. In our study, CA and BA at the start of GnRHa + GH treatment were 8.34 ± 0.44 years and 10.51 ± 0.91 years, respectively, which were older than those in the GnRHa group; however, there was no significant difference. Furthermore, the Tanner stage of the breast and pubic hair at the beginning of treatment showed no significant difference between the groups. These findings indicate that the age at the start of treatment is related to the growth rate rather than the degree of pubertal progression. Therefore, patients with a relatively late start of GnRHa treatment require close observation of their growth rate, and combined GH therapy may be considered.

A study on 448 Chinese children with CPP and early puberty divided participants into a control group (n = 118), GnRHa monotherapy group (n = 276), and combination therapy group (n = 54). In the combination therapy group, the height gain (FAH − initial PAH) and genetic height gain were 9.51 ± 0.53 cm and 4.00 ± 0.05 cm, respectively, which were significantly higher than those in the other groups^24^. In addition, when compared according to the GH treatment period in the combination therapy group, the height gain was significantly higher in the group starting after 6–12 months than in the group starting GH therapy at the same time as GnRHa treatment. Furthermore, in a meta-analysis published in China, the GH combination treatment significantly improved the height, PAH, and height SDS–BA. In addition, when the starting age of GH treatment was less than 10 years and the GH treatment period was 12 months or more, a significant improvement was confirmed in height, PAH, and height SDS–BA^11^.

In a previous study conducted of 20 girls with idiopathic CPP who received GnRHa monotherapy and GnRHa + GH treatment, pretreatment PAH and FAH were 155.5 ± 1.7 cm and 157.1 ± 2.5 cm, respectively, in patients treated with GnRHa alone. In the GnRHa + GH treatment group, pretreatment PAH was 152.7 ± 1.7 cm and FAH was 160.6 ± 1.3 cm. The height gain in the two groups was different, at 7.9 ± 1.1 cm and 1.6 ± 1.2 cm, respectively, which was significantly higher in combination treatment groups (*P* < 0.001)^25^. In a study of 35 girls with CPP published in Italy, the pretreatment PAH and FAH were 153.2 ± 5.0 cm and 161.2 ± 4.8 cm, respectively, in those treated with GnRHa + GH. In the groups treated with GnRHa alone, pretreatment PAH and FAH were 153.9 ± 3.8 cm and 156.6 ± 5.7 cm, respectively. The height gain when treated with GnRHa + GH was 12.7 ± 4.8 cm, which was higher than that in the GnRHa group, 2.3 ± 2.9 cm^26^. In the current study, the duration of GH treatment was 2.04 ± 0.90 years and the period between GnRHa and GH treatment was 6.59 ± 7.77 months. The height gain (FAH–initial PAH) was 9.22 ± 6.03 cm in patients treated with GnRHa + GH, which was significantly higher than the 4.72 ± 5.01 cm in those treated with GnRHa alone (*P* < 0.001). The ΔPAH in the GnRHa + GH group was 13.66 ± 6.39 cm, which was significantly higher than the GnRHa group (*P* = 0.005). This indicates a large progression in PAH in the combination treatment group and an improvement of height potential with GnRHa + GH treatment.

Previous studies showed that young CA at the time of diagnosis, height, height SDS, and PAH at the start and end treatment influence FAH after GnRHa monotherapy in girls with CPP^6,27–29^. However, studies on factors affecting FAH in GnRHa + GH treatment in CPP are scarce. Fu et al.^24^ reported that PAH, BA, and TH demonstrated positively affected adult height, similar to our study. In a meta-analysis study, younger age at initial treatment (< 10 years old) and longer GH treatment duration (> 12 months) were correlated with better outcome^11^. Therefore, it is suggested that combined GH therapy for 12 months or more may be required at a young age if GH treatment is required in patients with CPP. In our study, the factors influencing height gain in girls treated with GnRHa + GH were the PAH and BA at start of treatment. Contrary to expectations, GH treatment periods and dose were not identified as factors affecting gain in FAH. These differences may be explained by the differences in ethnic background and sample size. Among girls with CPP, the combined GnRH and GH treatment are generally well tolerated. Wang et al.^11^ analyzed 464 patients from 14 studies receiving GnRH + GH treatment. There were no obvious abnormalities in bone density, BMI, liver, and kidney function, thyroid function, and glucose metabolism. In another meta-analysis, no significant adverse effects were observed during treatment or after discontinuation^30^.

The current study had several limitations. First, this study was a retrospective single-center study; therefore, further large prospective studies are required. Second, the number of CPP patients treated with combined GH is relatively small. In Korea’s healthcare system, GH treatment for patients with CPP is expensive because it is not covered by medical insurance. The financial burden on the guardian is increased, so GH treatment is limited in patients with CPP. Thus, financial status may be a factor influencing our findings. In addition, it was difficult to perform blood tests such as IGF-1 due to high costs during GH treatment, so the degree of IGF-1 increase could not be confirmed in this study.

In conclusion, this study demonstrated the GnRHa + GH treatment is effective for girls with CPP. The gain in the FAH compared to PAH at the start of treatment was significantly higher with GnRHa + GH treatment when compared with GnRHa monotherapy. The combined GH therapy should not be recommended as routine treatment in the international clinical guideline for the management of CPP^13,31^. However, when the growth rate slowed down during GnRHa treatment and the expected adult height was very short, a combination of GH and GnRHa treatment could be taken into consideration.

## Data Availability

The datasets analyzed during the current study are available from the corresponding author on reasonable request.
